# KIF11 Promotes Proliferation of Hepatocellular Carcinoma among Patients with Liver Cancers

**DOI:** 10.1155/2021/2676745

**Published:** 2021-01-04

**Authors:** Zhan-Dong Hu, Ying Jiang, Hong-Mei Sun, Jing-wen Wang, Li-Li Zhai, Zhi-Qi Yin, Jun Yan

**Affiliations:** ^1^Department of Pathology in Tianjin First Central Hospital, Number 24, Convalescent Road, Nankai, Tianjin 300192, China; ^2^Department of Clinical Laboratory in Tianjin First Central Hospital, Number 24, Convalescent Road, Nankai, Tianjin 300192, China; ^3^Department of Out-Patient in Tianjin First Central Hospital, Number 24, Convalescent Road, Nankai, Tianjin 300192, China

## Abstract

**Background:**

Hepatocellular carcinoma (HCC) lacks effective treatments and has a poor prognosis. Therefore it is needed to develop more effective drug targets. Kinesin family member 11 (KIF11) has been reported to affect the progression of several cancers, and its high expression associates with the prognosis of patients. However, the relevant mechanisms of KIF11 in HCC progression have not been studied.

**Method:**

Through the cancer genome atlas (TCGA) database and immunohistochemical (IHC) staining of patients' specimens, we determined that KIF11 was highly expressed in HCC tissues and associated with prognosis. We established a KIF11 stably depleted hepatoma cell line, through cell-cloning experiments and cell counting kit-8 (CCK-8) assays to detect the effects on proliferation *in vitro*. The role of KIF11 in promoting cell proliferation was verified in mice.

**Result:**

The expression of KIF11 was negatively correlated with the overall survival (OS) and disease-free survival (DFS) and positively correlated with tumor size of HCC patients. KIF11 depletion inhibits cell proliferation and tumor growth *in vitro* and *in vivo. Conclusion*. KIF11 can be used as a positive correlation marker for HCC prognosis and served as a potential therapeutic target.

## 1. Introduction

Hepatocellular carcinoma (HCC) is the fifth most frequently diagnosed cancer and the second most common cause of cancer death worldwide, especially in South Africa and East Asia [1–6]. In 2010, new cases of HCC were up to 24,120. Unfortunately, the number of HCC patients has been rising, increasing to 42,030 new cases in 2012 in the U.S. Most patients with HCC were diagnosed at the advanced stage, and a minority patients were diagnosed at an early stage [2, 7, 8]. So, the prognosis of HCC patients was still poor. The mortality rate of HCC has also obviously increased, with 18,910 deaths in 2010 and 31,780 deaths in 2019, accounting for 5.2% of all cancer deaths in 2019 [9, 10]. Hepatocellular carcinoma (HCC) accounts for 90% of primary hepatic malignancies and is the most common form of primary liver cancer [11, 12]. The surgical therapy of HCC is not effective. For HCC patients with high metastasis, chemotherapy is the most common treatment. However, traditional chemotherapy has poor efficacy with obvious side effects [13, 14]. Therefore, we need to develop new molecular biomarkers and targets that indicate prognosis and improve treatment.

Kinesin family member 11 (KIF11, also known as EG5) consists of 1056 amino acids encoding a motor protein that belongs to the kinesin-like protein family. The kinesin superfamily (KIF) is a highly conserved motor protein subdivided into 14 families (Kinesin 1–14A/B) [15–17]. Their domains bind and step across microtubules by transforming the chemical energy of ATP into mechanical forces [18, 19]. KIF proteins play vital roles in cellular functions, including mitosis and intracellular trafficking of vesicles and organelles [18, 20]. Due to the overexpression of KIF proteins, such as KIF11, additional forces are generated during mitosis, leading to premature separation of sister chromatids and unequal chromosome distribution, which may further result in aneuploidy of progeny cells [18]. The genetic instability due to KIF11 defects leads to cancer progression, for example by increasing the development of invasion and metastasis [21]. KIF11 is broadly expressed in lymph node, bone marrow, and other normal tissues. Recent researches reported that KIF11 was overexpressed in several tumors and associated with the poor prognosis, such as prostate cancer [22], gastric cancer [23], non-small cell lung cancer [24], oral cancer [25], meningioma [26], hepatic carcinoma [27], and pancreatic tumor [28].

In this study, firstly, we revealed the expression of KIF11 in hepatocellular carcinoma through analyzing TCGA database and IHC staining of patient specimens. And then, we clarified the relationship between the expression of KIF11 and the prognosis and clinicopathological characteristics of hepatocellular carcinoma. Finally, we use *in vivo* and *in vitro* experiments to verify the expression of hepatocellular carcinoma after knockdown KIF11. Our main purpose is to provide new targets for the diagnosis and treatment of HCC.

## 2. Materials and Methods

### 2.1. Bioinformatics and Statistical Analysis

To explore the different expressions of KIF11 in HCC and paired/unpaired adjacent normal liver tissues, we searched in TCGA database (https://www.cancer.gov/). For overall survival and disease-free survival analysis, we used the web tool of GEPIA (http://gepia.cancer-pku.cn/), median for group cutoff, 95% confidence interval.

### 2.2. Cell Lines and Tumor Samples

Hep 3B cells were cultured in Eagle's Minimum Essential Medium (30-2003, ATCC, Manassas, USA), and SNU-475 cells were culture in RPMI-1640 medium (30-2001, ATCC, USA), respectively. All cells were maintained with 10% fetal bovine serum (FBS, BioLegend, Beit HaEmek, Israel) at 37°C with 5% CO_2_. Liver tumor samples and adjacent normal tissues were collected from patients undergoing surgeries at our hospital. All samples in this research were approved by the patients and Ethics Committee of Hospital.

### 2.3. Immunohistochemical (IHC) Staining

All tissues were formalin-fixed and paraffin-embedded, and then, the tissues were cut into 4 *μ*m sections. After being deparaffinized and rehydrated, antigen retrieval was performed in citrate buffer, blocked with goat serum. After being washed, the tissues were incubated with primary antibodies (KIF11 1 : 100 dilution, ab61199) overnight at 4°C. Then, the sections were washed with PBS. Then, the sections were incubated with secondary antibody-HRP conjugate at room temperature. Finally, the sections were stained with DAB for 5 min and counterstained with hematoxylin.

### 2.4. Lentivirus-Mediated shRNA Interference

The sequence of siRNA-KIF11 (sense 5′-CCACGTACCCTTCATCAA-3′) and negative control siRNA (sense 5′-UUCUCCGAGCGUGUCACGUTT-3′) were cloned into the PLKO.1 vector. Then, the lentivirus vectors cotransfected into HEK-293T cells with the packing vector. After 72 hours, the lentivirus was collected from the supernatant and added to target tumor cells. After 3 days, 2 *μ*g/mL puromycin (P8230, Solarbio) was used to screen positive cells.

### 2.5. Quantitative RT-PCR

Total RNA was extracted using the TRIzol reagent and made into cDNA using reverse transcription kit. Then, quantitative PCR was performed using the SYBR MasterMix (4913850001, Roche, Basel, Switzerland), ABI 7900HT, according to the manufacturer's instructions, and then the difference was calculated using the 2^–*ΔΔ*CT^. KIF11 primer is as follows: forward 5′-GAA CAATCATTAGCAGCAGAA-3′ and reverse 5′-TCAGTATAGACA CCACAGTTG-3′ [27]. *β*-Actin primer is as follows: forward primer 5′-TAATCTTCGCCTTAA TACTT-3′ and reverse primer 5′-AGCCTTCATACATCTCAA-3′ [27].

### 2.6. Western Blot

All proteins from cells or tumors were resolved by SDS-PAGE gel (P1200, Solarbio, Beijing, China) and transferred onto PVDF membranes (LC2002, Thermo, Waltham, US). Then, the membranes were blocked in 5% fat-free milk in TBST for 1 hour at room temperature and then washed, and primary antibodies were incubated at 4°C overnight. After being washed, the PVDF was incubated with horseradish peroxidase-conjugated secondary antibodies. The bands were visualized by ECL reagent (32106, Thermo). Antibodies are as follows: KIF11 (1 : 1000 dilution, ab61199, Abcam, Cambridge, UK); anti-*β*-actin (1 : 1000 dilution, ab5694, Abcam, Cambridge, UK).

### 2.7. Colony Formation Assay

Cells were seeded into 6-wells with 2 mL complete medium, containing 500 cells. After 2 weeks of culture in a cell culture incubator, 4% paraformaldehyde was used for the fixation of the cells and then stained with 1% crystal violet. Then, the ImageJ software was used to calculate the number of cell clones.

### 2.8. CCK-8 Assay

1 × 10^4^ control tumor cells and sh-KIF11 tumor cells with 100 *μ*L complete medium were seeded into 96-wells. After 3 days incubation, cell viability was quantified using CCK-8 reagent (B34302, Bimake, China). Briefly, 10 *μ*L of CCK-8 reagent was added to each well according to the instructions and cultured at 37° C for 3 hours, and the absorbance was measured at 490 nm wavelength using a microplate reader.

### 2.9. Xenograft Mouse Tumors

A total of ten 6-8-week-old nude-BALB/c mice were randomly divided into sh-KIF11 group and control group. 5 × 10^6^ SNU-475 cells (sh-KIF11 or control cell) embedded into 100 mL Matrigel were injected into the flank of mice. The tumors volume was recorded every 3 days (volume = length × width^2^ × 0.5). After 30 days, the mice were sacrificed, and the tumors were removed for analysis.

### 2.10. Statistics

GraphPad 6.0 was used for statistical analysis. All results in this study were represented as mean ± SEM. The Kaplan-Meier (KM) method was used to detect the relationship between the expression and postoperative survival time. The correlations between clinical features and KIF11 expression were analyzed through the *χ*^2^ analysis. Student's *t*-test was used for statistical comparisons. ∗ indicated *p* < 0.05, which was also considered a statistically significant difference, and also, ∗∗ indicated *p* < 0.01, and ∗∗∗ indicated *p* < 0.001.

## 3. Results

### 3.1. KIF11 Is Associated with the Poor Prognosis of HCC Patients

**Figure 1:**
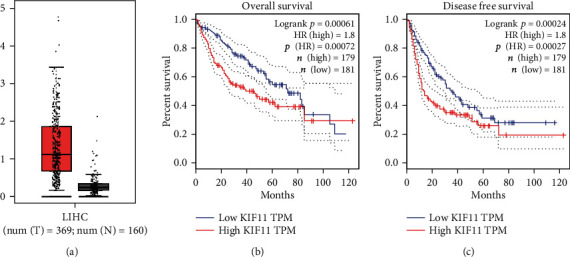
KIF11 expression and the correlation with the prognosis in TCGA database. (a) KIF11 expression levels between HCC tissues (*n* = 369) and normal liver tissues (*n* = 160) in TCGA database. (b) Overall survival (OS) and disease-free survival (DFS) of patients with different expression levels of KIF11. The OS and DFS were analyzed using the GEPIA web tool.

**Figure 2:**
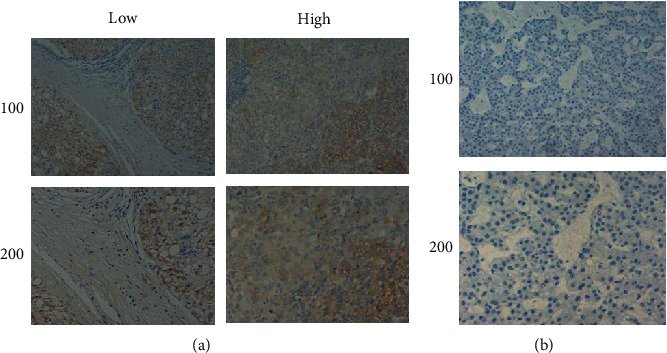
IHC staining for KIF11 in HCC and normal liver tissues. (a) KIF11 was high expression in HCC tissues compared with normal liver tissues (b) and mainly expressed in the cytoplasm.

To explore the relationship between KIF11 expression and HCC progression, we first analyzed the data in TCGA database, showing that KIF11 expression in HCC tissues was significantly higher than normal liver tissue (Figure 1(a)). Patients with high KIF11 expression had poor overall survival (OS, *p* = 0.00072) and disease-free survival (DFS, *p* = 0.00027, Figure 1(b)). We collected the clinical case information from 70 patients with HCC and used IHC staining to detect KIF11 expression in the patient's tissues and found that KIF11 was positively correlated with tumor size (*p* = 0.016, Table 1, Figures 2(a) and 2(b)). In conclusion, we thought KIF11 was associated with poor prognosis in HCC patients.

### 3.2. Establish Stable Cell Lines of Knockdown KIF11 Using shRNA Plasmids

**Figure 3:**
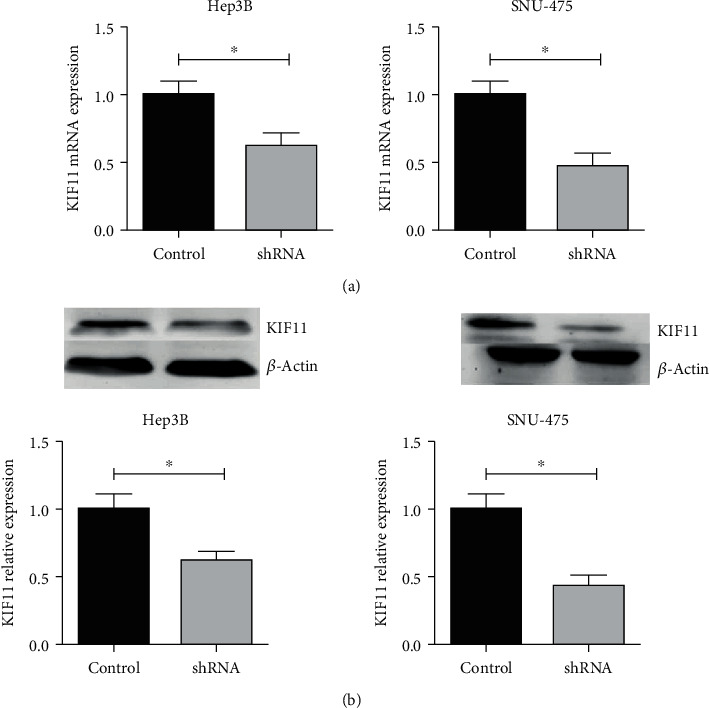
Lentiviral-mediated shRNA interference with KIF11 expression. After adding virus-mediated shRNA for 3 days, the total mRNAs and proteins of the cells were extracted, and the interference effect was detected by qPCR (a) and western blot (b). Adding puromycin to cells for screening positive cells.

To further investigate the regulatory mechanism and effects of KIF11 on HCC cells, we selected two HCC cell lines, Hep3B and SNU-475, using lentivirus-mediated shRNA to interfere with KIF11 expression. A stably KIF11-depleted cell line was established by screening for puromycin to study the mechanism by which KIF11 affected tumor progression. Knockdown efficiency was verified at mRNA (Figure 3(a)) and protein levels (Figure 3(b)) using QPCR and western blot, respectively.

### 3.3. Interfering with KIF11 Inhibits Cell Proliferation in Hep3B and SNU-475 Cells

**Figure 4:**
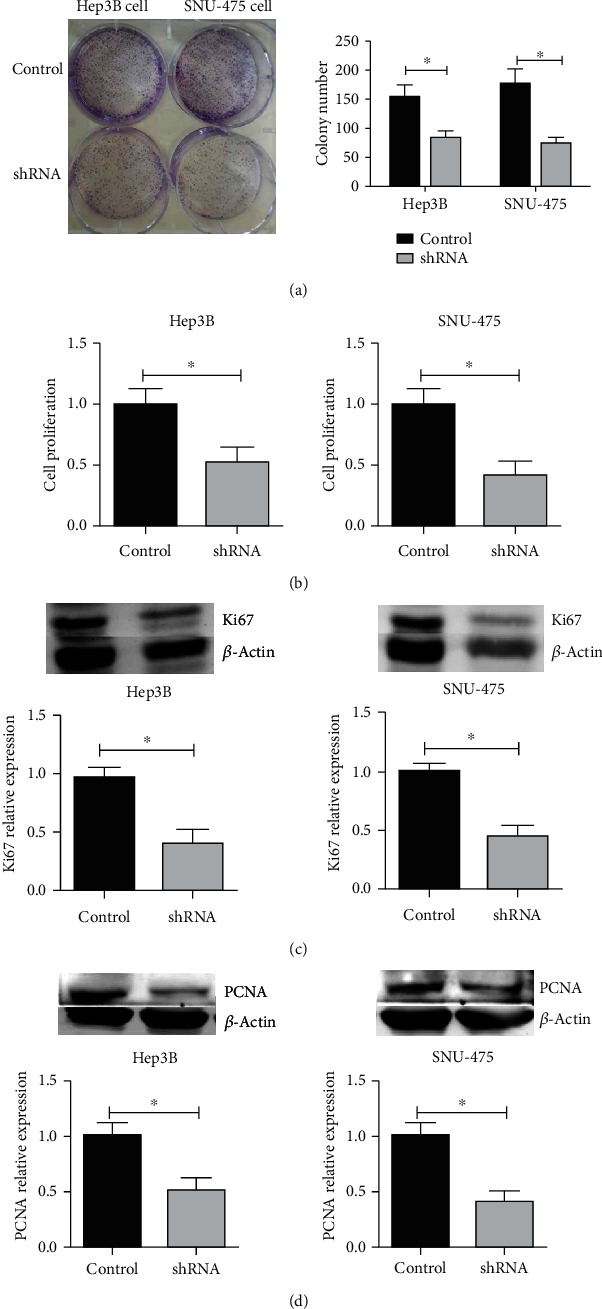
Interfering with KIF11 inhibits the proliferation of Hep3B and SNU-475 cells. (a) The cell colony formation assays and CCK-8 assay (b) were performed to detect cell proliferation after knockdown KIF11 in Hep3B and SNU-475 cells. Western blot showed that after knocking down KIF11, Ki-67, and PCNA expressions were decreased (c, d).

Then, we investigated whether KIF11 modulated cancer cell proliferation in HCC cells. The colony formation assays were performed to detect cell proliferation with normal cancer cells and KIF11 depletion cells, suggesting that KIF11 depletion suppressed cell colony formation in Hep3B and SNU-475 cell lines (Figure 4(a)). Similar results were shown in CCK-8 assays (Figure 4(b)). Ki-67 and PCNA are two proliferation-related proteins, and their expression levels can reflect cell proliferation degree. Western blot assays showed that Ki-67 and PCNA expression was decreased after knockdown of KIF11 (Figures 4(c) and 4(d)).

### 3.4. Knockdown KIF11 Decrease HCC Proliferation and Tumor Growth *In Vivo*

**Figure 5:**
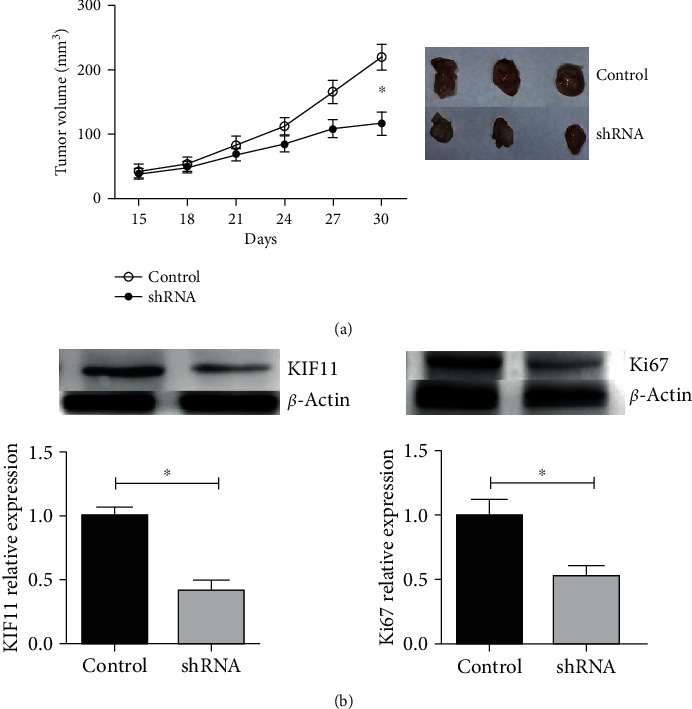
Knockdown of KIF11 decreases HCC proliferation *in vivo*. (a) After tumor inoculation, the volume of the tumor was observed and recorded every 3 days. After 30 days, the mice were sacrificed, and the tumors were taken out. (b) The protein expression of KIF11 and Ki-67 were decreased in tumor tissues from the KIF11 depletion group.

To confirm the functions of KIF11 *in vivo*, 5 × 10^6^ SNU-475 cells embedded into Matrigel were injected in the flank of 6-8-week-old nude-BALBc mice. The tumor volume reached a level of macroscopicity after 15 days of implantation. The size of the tumor volume was measured every 3 days, and after 2 addition weeks, the control tumor volume reached approximately 200 mm^3^, whereas sh-KIF11 only reached 110 mm^3^ (Figure 5(a)). To further characterize the control cells and sh-KIF11 cells, the KIF11 and proliferation marker, Ki-67, were detected using western blot in tumors, showing that the expression of Ki-67 and KIF11 were lower than control cells (Figures 5(b) and 5(c)). In short, knockdown KIF11 also resulted in reduced cell proliferation *in vivo*.

## 4. Discussion

HCC is a devastating disease with a poor prognosis in all major malignancies. There is an urgent need to identify molecular markers associated with the pathogenesis of the disease for more timely and effective diagnosis and treatment. Importantly, we found KIF11 is a highly expressed protein by searching the TCGA database and IHC stain of clinical HCC samples. In addition, we also uncovered a strong correlation between KIF11 expression and clinical pathological parameters, such as OS, DFS, tumor size, and malignancy of HCC. Liu et al. previously evaluated mRNA and protein expression levels of KIF11 in 26 freshly frozen HCC tissues and normal samples, which showed that mRNA and protein of KIF11 were significantly higher than matched normal live tissues. Moreover, the KIF11 expression level was strongly associated with TNM stage and liver cirrhosis, and Cox regression analysis showed that KIF11 was an independent factor for overall survival [27]. IHC stain showed that KIF11 protein was mainly expressed in cell cytoplasm, consistent with previous studies [29]. Due to poor patient compliance and since we have not established a complete follow-up system, we only did a single variable analysis, and we have not done a follow-up for survival analysis, etc. We strive to make multifactor analysis in future research to make the results more diversified and more credible.

In other types of cancers, KIF11 also acts as an oncogene, and knockdown of KIF11 inhibits tumor growth [29–31]. Our experiments implicated knockdown of KIF11 by lentiviral-mediated shRNA inhibits tumor cell growth *in vitro* and *in vivo*.

As we know, KIF11 plays an essential role in the formation and maintenance of bipolar spindles during mitosis as a microtubule-based motor. In addition to its role in mitosis, more studies have shown other biological functions. In the immune system, KIF11 acts with Tat to promote apoptosis in CD4^+^ cells [32]. In pancreatic cancer cells, Liu et al. showed that KIF11 promoted cell proliferation dependence ATPase, and ATPase-dead mutant inhibited cell proliferation [28]. Higher expression of KIF11 disturbed bipolar spindle formation, triggering multipole spindle assembly and weakening chromosome segregation. Additionally, the overexpression of KIF11 causes genomic instability in cells and prompts abnormal proliferation in mice [33].

Research and development of small molecules targeting KIF11 are undergoing clinical trials [34]. We found that KIF11 is overexpressed in HCC tissues, and KIF11 shRNA significantly inhibits proliferation and tumor formation of HCC cells, suggesting that KIF11 may serve as an effective target for the treatment of HCC.

In this study, we showed that KIF11 was highly expressed in HCC tissues and associated with the prognosis. Our results in the present study have uncovered underlying mechanisms how the expression of KIF11 is involved in the progression of HCC. The exact role of KIF11 in the development of liver cancer needs to be clarified in further research.

## 5. Conclusions

In short, we firstly showed that elevated KIF11 expression was significantly correlated with the poor clinical outcome in HCC. Then, our *in vitro* experiments proved that interfering with KIF11 inhibits cell proliferation in Hep3B and SNU-475 cells. Similarly, knockdown KIF11 inhibited the proliferation of HCC cells and tumor growth *in vivo*. Due to limited energy and funds, we will conduct external validation to verify the above conclusion in future research. And we speculate that KIF11 may be a potential target for the treatment of HCC in the future.

## Figures and Tables

**Table 1:** Relationships of KIF11 expression and clinicopathological characteristics in 70 patients with HCC.

Feature	All *n* = 70	KIF11 expression		*χ* ^2^	*p*
		Low	High		
		*n* = 36	*n* = 34		
Age (year)				0.489	0.484
<55	38	21	17		
≥55	32	15	17		
Gender				0.477	0.490
Male	40	22	18		
Female	30	14	16		
Tumor grade				2.979	0.084
Low	30	19	11		
High	40	17	23		
Tumor size				5.826	0.016∗
≥5 cm	48	20	28		
<5 cm	22	16	6		
AFP (ng/mL)				0.278	0.598
<50	41	20	21		
≥50	29	16	13		

## Data Availability

The dataset supporting the conclusions of this article is included within the article.

## Abbreviations

KIF11: Kinesin family member 11
IHC: Immunohistochemistry
DAB: 3,3-Diaminobenzidin
HRP: Horseradish peroxidase
PCNA: Proliferating cell nuclear antigen
PBS: Phosphate-buffered saline
PAGE: Polyacrylamide gel electrophoresis
QRT-PCR: Quantificational real-time polymerase chain reaction
shRNA: Short hairpin RNA.
